# AMP Deaminase 3 Deficiency Enhanced 5′-AMP Induction of Hypometabolism

**DOI:** 10.1371/journal.pone.0075418

**Published:** 2013-09-16

**Authors:** Isadora Susan Daniels, William G. O′Brien, Vinay Nath, Zhaoyang Zhao, Cheng Chi Lee

**Affiliations:** Department of Biochemistry and Molecular Biology, University of Texas Health Science Center, Houston, Texas, United States of America; University of Colorado Denver, United States of America

## Abstract

A hypometabolic state can be induced in mice by 5′-AMP administration. Previously we proposed that an underlying mechanism for this hypometabolism is linked to reduced erythrocyte oxygen transport function due to 5′-AMP uptake altering the cellular adenylate equilibrium. To test this hypothesis, we generated mice deficient in adenosine monophosphate deaminase 3 (AMPD3), the key catabolic enzyme for 5′-AMP in erythrocytes. Mice deficient in AMPD3 maintained AMPD activities in all tissues except erythrocytes. Developmentally and morphologically, the *Ampd3^−/−^* mice were indistinguishable from their wild type siblings. The levels of ATP, ADP but not 5′-AMP in erythrocytes of *Ampd3^−/−^* mice were significantly elevated. Fasting blood glucose levels of the *Ampd3^−/−^* mice were comparable to wild type siblings. In comparison to wild type mice, the *Ampd3^−/−^* mice displayed a deeper hypometabolism with a significantly delayed average arousal time in response to 5′-AMP administration. Together, these findings demonstrate a central role of AMPD3 in the regulation of 5′-AMP mediated hypometabolism and further implicate erythrocytes in this behavioral response.

## Introduction

It is widely known that in response to severe metabolic stress, some mammals enter a period of torpor or hibernation, physiological states of severely reduced metabolic rates, to survive [1]. The biochemical and physiological processes by which torpor is induced and maintained are poorly understood. We have previously shown that the natural metabolite adenosine monophosphate (5′-AMP), when given in sufficient dosages to mice or other mammals, can induce a state of transient hypometabolism [2] [3]. During this hypometabolic state, the animal's core body temperature (T_b_) can be safely and readily reduced from euthermia. If maintained at an ambient temperature (T_a_) of 15 °C, the T_b_ of mice could be reduced to 1–2°C above T_a_ for 6–9 h and the process is reversible [3]. Questions have been raised as to whether this hypometabolic state associated with the T_b_ decline induced by 5′-AMP is torpor [4]. Recently, the deep hypothermia behavior associated with 5′-AMP induced hypometabolism has been recognized as a form of induced torpor by investigators who have studied natural torpor behaviors [5].

Our previous studies implicated erythrocytes as the primary cellular target of 5′-AMP [3]. We demonstrated that 5′-AMP uptake by erythrocytes alters the cellular adenylate ratios, disrupts glycolysis, and results in a rise in glycolytic intermediates, including the metabolite 2,3-bisphosphoglycerate (2,3-BPG). It is well established that 2,3-BPG reduces hemoglobin affinity for oxygen allosterically [6]. The observed rapid decline in the apparent consumption of oxygen, a key feature observed in mammals given 5′-AMP, is not inconsistent with reduced oxygen affinity of the hemoglobin in the erythrocytes. We proposed that it is the alteration in erythrocyte adenylate ratios by 5′-AMP uptake that is the underlying biochemical mechanism initiating the physiological responses that result in the hypometabolic state.

Cellular catabolic pathways for 5′-AMP could either occur through its deamination to inosine monophosphate (IMP) by AMP deaminase (AMPD), or its dephosphorylation to adenosine [7]. Cellular adenosine can either be phosphorylated to 5′-AMP by adenosine kinase or deaminated to inosine by adenosine deaminase (ADA). However, the K_m_ for adenosine of ADA is more than an order of magnitude larger than that of adenosine kinase, favoring adenosine phosphorylation to 5′-AMP over deamination to inosine by ADA [7]. Thus, the majority of cellular 5′-AMP is catabolized by AMP deaminases. In mice, there are three known isozymes of AMP deaminase that are encoded by three different genes: the muscle isoform (AMPD1), the liver isoform (AMPD2) and the erythrocyte isoform (AMPD3) [8]. Many tissues express various levels of all three AMPD isozymes. However, AMPD3 is the only known isozyme present in erythrocytes [9]. Therefore, we reasoned that disrupting AMPD3 function should alter the catabolism of 5′-AMP specifically in erythrocytes. An *Ampd3^−/−^* mouse would allow for the examination of the erythrocyte's role in mediation of 5′-AMP induced hypometabolic behavior. Hence, we undertook a study to disrupt the gene for AMPD3 in mice. Our findings demonstrate that the loss of AMPD3 significantly enhanced the efficacy of injected 5′-AMP to induce a hypometabolic response in mice.

## Materials and Methods

### Creation of AMPD3 deficient mice

ES cells (C57Bl/6 background) containing the vector to generate the AMPD3 deficient mice were purchased from the NIH Knockout Mouse Project (KOMP) [10], via the KOMP repository at the University of California, Davis. The ES cells from KOMP were amplified and verified using a PCR genotyping protocol provided by the supplier. Chimeric mice were generated using the ES cells at the Transgenic and Stem Cell Service Unit of the Brown Institute of Molecular Medicine, Houston, TX. Briefly, the ES cells were injected into blastocysts of pseudopregnant C57Bl/6 females to generate chimeric offspring that carry the *Ampd3* gene targeted allele. Eight male chimeras (C57Bl/6 wild type, Balbc *Ampd3* knockout) were generated, and all were mated with C57Bl/6 wild type females purchased from Harlan Laboratories, Houston, TX. Of the eight chimeras, two were able to produce germline transmission to create *Ampd3^+/−^* mice, which were then mated to produce the *Ampd3^+/+^*, *Ampd3^+/−^* and *Ampd3^−/−^* mice used in experiments.

All animal studies were carried out following the recommendations in the Guide for the Care and Use of Laboratory Animals of the National Institutes of Health. The animal research protocols (HSC-AWC-09-138 and HSC-AWC-13-012) were approved by the Animal Welfare Committee (AWC), the Institutional Animal Care and Use Committee (IACUC) for the University of Texas Health Science Center at Houston.

### In vivo Metabolic Rate Measurement

Metabolic rates of live mice assessed by their oxygen consumption (VO_2_) and/or carbon dioxide production (VCO_2_) were measured in a Comprehensive Lab Animal Monitoring System (CLAMS) from Columbus Instruments, Columbus, OH. Each animal was housed individually in an airtight cage with gases going in and out of the cage measured by high-speed gas sensors on an Open Circuit Oxymax Calorimeter, (Columbus Instruments, Columbus, OH). The airflow into and out of the cage was calibrated against a known gas mixture before each experiment. Samples were taken every minute, cage by cage, with one extra minute for re-calibration at every four samplings. The animals had access to food and water ad libitum during the entire study.

### Reverse Transcription Polymerase Chain Reaction (RT-PCR)

Heart tissues were harvested from WT and *Ampd3^−/−^* mice. Total RNA was isolated using Trizol (Invitrogen), following the vendor's instructions. First-strand cDNA synthesis was carried out using 1 µg of total RNA and oligo (dT) primer with SuperScript II reverse transcriptase (Invitrogen). Then the first strand cDNA was used for PCR using a kit (Roche USA), with forward primer, 5′-GTATGCACGGCTTGCCTACCAC; and reverse primer, 5′- AATAAGGGTCTTCCTGGGGCAGT. The cycling condition was: 95°C for 2 min, followed by 30 cycles of 95 °C for 30 sec, 59°C for 30 sec, and 72°C for 1 min, followed by incubation at 72 °C for 5 min. The resultant PCR products were visualized on a 1.5% agarose gel in 1xTAE buffer [11].

### AMP Deaminase Assay

AMP deaminase activity was assayed as previously described [12]. Briefly, AMPD activity was quantified by measuring formation of IMP when protein samples were added to reaction mixtures with saturating amounts of 5′-AMP. Each 100 µl reaction mixture contained 20 µg soluble cell lysate, 25 mM imidazole, pH 7.0, 0.2 mg/ml bovine serum albumin (BSA) and 150 mM potassium chloride. The addition of 20 mM 5′-AMP at 37°C initiated the enzymatic assay. A 50 µl aliquot was removed at 60 min and frozen at −80°C. Samples were then analyzed by high performance liquid chromatography (HPLC) (Alliance 2695 Separations Module with 2998 Photodiode Array Detector, Waters, Millipore Corp.) using a 5 µm C18 reversed phase column and elution by a mobile phase of 20 mM ammonium phosphate, pH 5.1 with a methanol gradient of 0–4 min 0% methanol, 4–6 min 0–8% methanol, 6–8 min 8–20% methanol and 8–18 min 20% methanol.

### Analysis of adenine nucleotides in erythrocyte lysates

Erythrocytes were isolated from whole blood by removing serum and washing 3 times in ice-cold phosphate-buffered saline. Cells were lysed by freeze/thaw and then extracted with 3 volumes of ice-cold 70% methanol overnight. The supernatant was removed after centrifugation at 12,000×g at 4°C for 10 min. The supernatant was evaporated to dryness and then resuspended in 200 µl mobile phase solvent. The protein pellet was used to determine protein concentration. Nucleotides in the extracts were separated by HPLC (Alliance 2695 Separations Module with 2998 Photodiode Array Detector, Waters, Millipore Corp.) using a 5 µm C18 reversed phase column (Sunfire, Waters, Millipore Corp.) with a mobile phase of 150 mM KH_2_PO_4_,150 mM KCl, pH 6.0, and a superimposed gradient using 15% acetonitrile (ACN) programmed as 0–20 sec 0% ACN, 20 s – 21 min 0–1.35% ACN, 24 min – 28 min 1.35–15% ACN, 29 min – 35 min 0% ACN. Nucleotides were quantified against standard curves of purchased standards (Sigma-Aldrich, St. Louis, MO).

### Blood Glucose Measurements

Mice were fasted overnight. Blood glucose was measured from whole blood with a One Touch Basic glucose meter (Life Scan, Inc).

### Data analysis

Standard statistical analyses are applied to assays of multiple samples yielding a value representing the mean ± standard error of the mean (SEM) [SEM = SD/(square root of sample size)]. The sample sizes (n) for each assay are shown in the corresponding figure legend. Asterisk (*) indicates statistical significance (*p*<0.05) in the two-tailed Students "t" test.

## Results

### Generation of *Ampd3^−/−^* Mice

**Figure 1:**
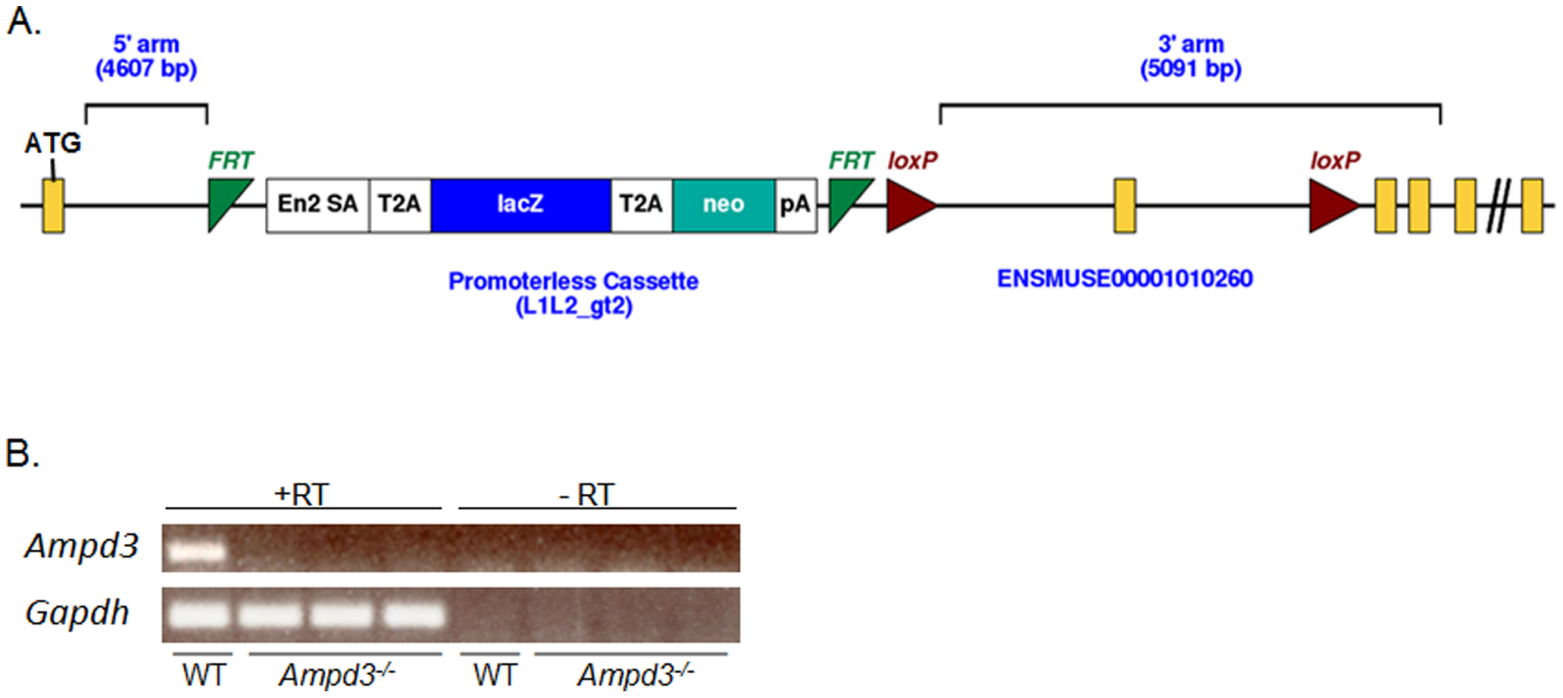
The “knockout-first” conditional construct for use at the *Ampd3* genomic loci. A. Recombination at the 5′ and 3′ homologous arms of the vector to the target sequences of the *Ampd3* gene allows the insertion of an en2-LacZ-neo cassette into a region spanning the intron between exon 5 and exon 6 of the *Ampd3* locus. With the “knockout-first” design, the 3′ end of exon 5, the first coding exon, is spliced into the splice acceptor (SA) site of the en2-LacZ-neo cassette in order to disrupt and destabilize the *Ampd3* transcript. B. RT-PCR showing that *Ampd3* mRNA from the heart of wild type (WT) mice is absent in *Ampd3^−/−^* heart tissue. “-RT” indicates the negative control where reverse transcriptase was lacking in parallel reactions.

To take advantage of the existing genetically engineered mouse resources in the scientific community, we searched for Ampd3 gene targeting projects from the KOMP catalog of mice and embryonic stem (ES) cells (C57Bl/6 background). An ES clone with *Ampd3* gene loci disrupted by a targeting vector, generated by The Sanger Center, was available. The vector applied the “knockout-first” conditional gene knockout technology to insert an en2-LacZ-neo cassette into an intron downstream from exon 5 of the AMPD3 gene locus (Figure 1A). With the “knockout-first” design, the initial targeted allele is predicted to result in a null allele by splicing the upstream exon of the targeted gene into a lacZ trapping element contained in the targeting cassette [13] [14]. By including a mouse En2 splice acceptor and the SV40 polyadenylation sequence in the trapping cassettes, this strategy has proven to be reliable in creating null alleles in mice. The outcome of the AMPD3 targeting is that the 3′ end of exon 5, containing the first ATG of the 14 coding exons, is spliced into the splice acceptor (SA) site of the en2-LacZ-neo cassette, disrupting and destabilizing the *Ampd3* transcript. As a result, there should be no functional *Ampd3* mRNA produced. These *Ampd3^−/−^* ES cells were used to generate the AMPD3 deficient mice.

Once the putative *Ampd3^−/−^* mice were generated, the animals were apparently developmentally normal based on gross morphological examination. In order to verify if the *Ampd3^−/−^* mice expressed *Ampd3* mRNA, *Ampd3* specific RT-PCR analysis was carried out with heart mRNA from wild type and *Ampd3^−/−^* mice. *Ampd3* is the dominant isoform in heart tissue and is highly expressed. As shown in Figure 1B, RT-PCR analysis showed the expected transcript band was present in wild type but not in *Ampd3^−/−^* mice. As a control, *Gapdh* transcripts were detected in all *Ampd3^−/−^* samples when cDNA was made (+RT), and were absent in all samples when cDNA was not made (-RT, the negative controls). The RT-PCR analysis shows that the *Ampd3^−/−^* but not the wild type animal heart samples were deficient in the *Ampd3* transcript.

### Absence of AMP Deaminase Activity in *Ampd3^−/−^* Erythrocytes

To further verify the RT-PCR findings, we then assayed AMP deaminase activity in various tissues obtained from siblings of wild type and *Ampd3^−/−^* genotypes. To assay for AMP deaminase activity, cell lysates prepared from various tissues obtained from wild type and *Ampd3^−/−^* mice were incubated with 5′-AMP and the amount of inosine monophosphate (IMP) formed was determined by HPLC. The enzymatic formation of IMP was then normalized to the amount of total lysate protein used in the reaction.

**Figure 2:**
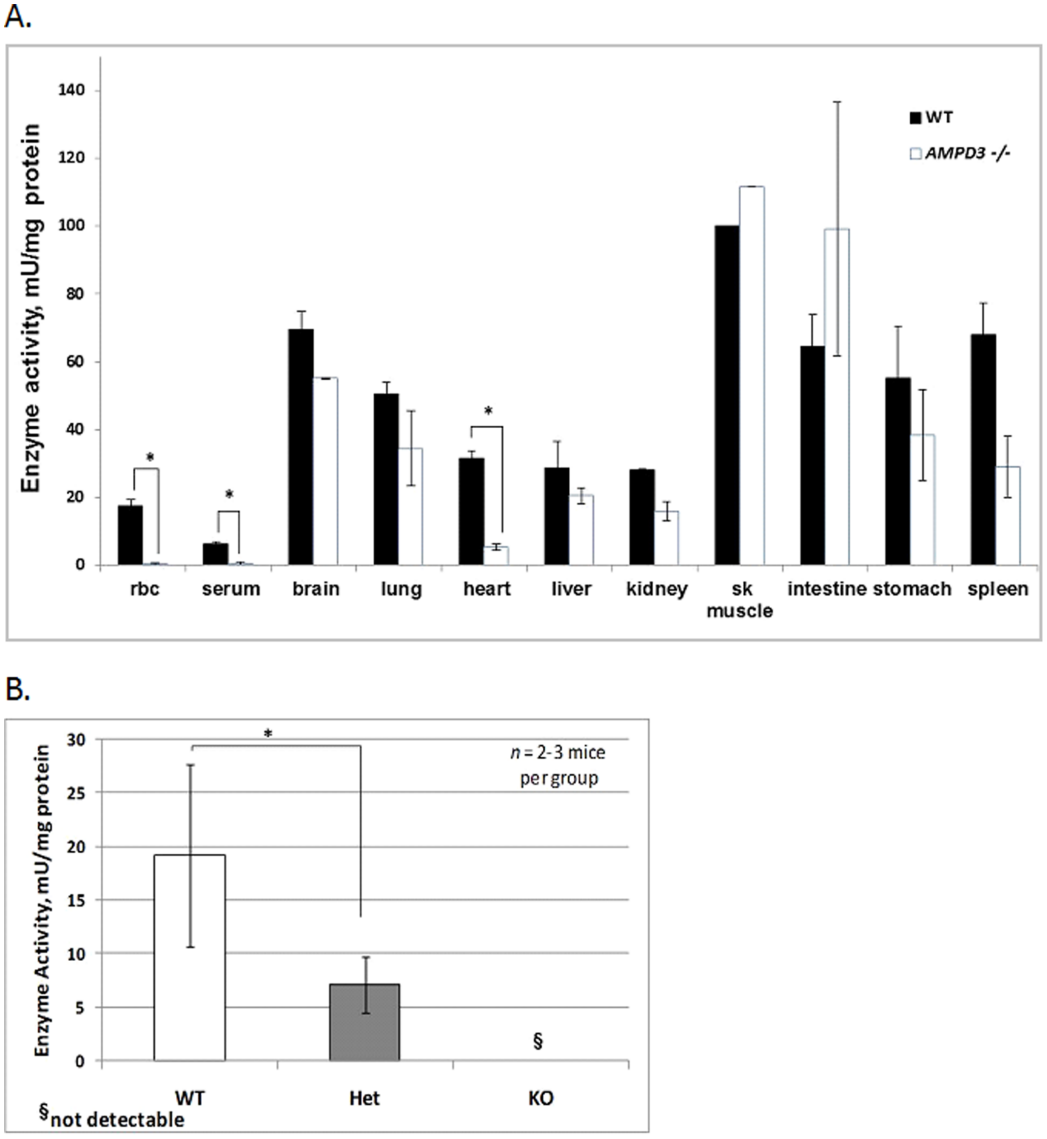
AMPD activity is not detectable in erythrocytes (rbc) and is reduced in the heart of *Ampd3^−/−^* mice. A. AMP deminase assays were carried out with cell lysates from the indicated organs. For all samples, except serum (WT n = 10; *Ampd3^−/−^* n = 5), heart (n = 8) and erythrocytes (WT n = 15; *Ampd3^−/−^* n = 7), the data represent the average of tissue samples (n = 3). B. The level of AMPD3 activities in erythrocytes from wild type, *Ampd3^+/−^* and *Ampd3^−/−^* mice. Error bars, mean ± SEM. T-test: *p<0.05.

In most tissues sampled, the levels of AMP deaminase activity did not differ significantly between wild type and *Ampd3^−/−^* mice (Figure 2A). This is expected since the other isoforms of AMP deaminases are present in varying levels in various tissues. However, there was no detectable AMP deaminase activity in erythrocyte lysates from *Ampd3^−/−^* mice in contrast to the wild type erythrocyte lysate. Consistently, there was also no detectable AMP deaminase activity in the serum fraction of blood obtained from *Ampd3^−/−^* mice. In the heart, where AMPD3 is a major isozyme, the AMP deaminase activity was significantly lower in *Ampd3^−/−^* mice when compared to wild type. The erythrocyte lysate of heterozygous *Ampd3^+/−^* mice had significantly less AMP deaminase activity than the wild type (Figure 2B). Together, these results demonstrate that there is no measurable AMP deaminase activity in the erythrocytes of *Ampd3^−/−^* mice.

### ATP and ADP are elevated in *Ampd3^−/−^* erythrocytes

**Figure 3:**
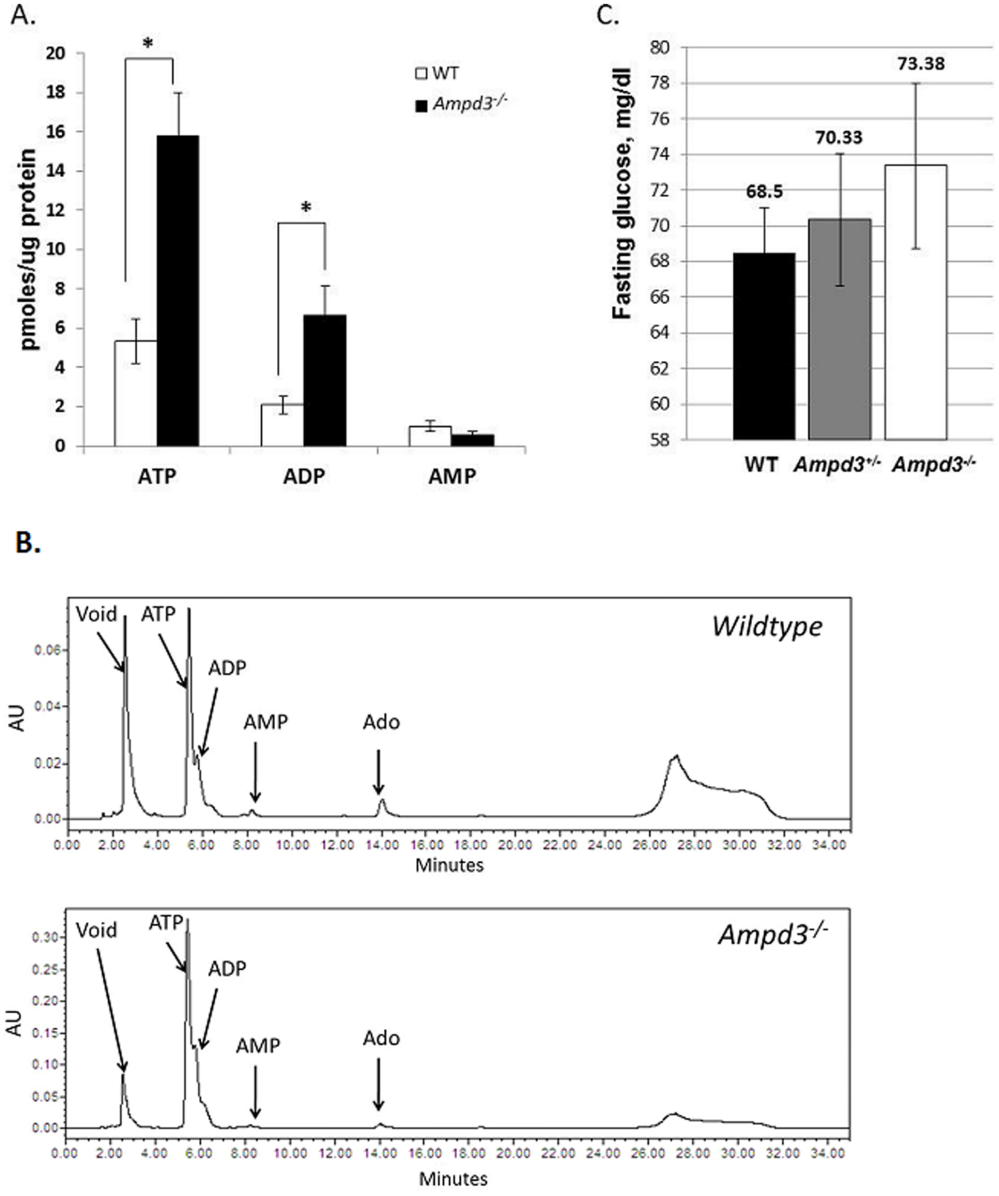
Erythrocytes of *Ampd3^−/−^* mice have elevated levels of ATP and ADP but normal levels of AMP. A. Nucleotides from methanol extracts of erythrocyte lysates from wild type (n = 4) and *Ampd3^−/−^* (n = 4) were separated by HPLC and quantified by calibration with standards. B. Representative HPLC chromatograms from wild type and *Ampd3^−/−^* erythrocyte nucleotide extracts. C. Fasting glucose levels in *Ampd3^−/−^* (n = 16) and *Ampd3^+/−^* mice (n = 9) and WT (n = 14) mice. Error bars, mean ± SEM. T-test: *p<0.05.

In humans, the loss of AMP deaminase 3 is associated with an increased steady-state level of ATP in erythrocytes [15]. Therefore, we measured whether the erythrocytes of *Ampd3^−/−^* mice displayed a similar increase in ATP. HPLC analysis was performed to quantify the levels of ATP, ADP and AMP in methanol extracts of erythrocytes obtained from wild type and *Ampd3^−/−^* mice (Figure 3A, 3B).

Adenine nucleotides levels were quantified against calibration standards for ATP, ADP and AMP, and normalized to the protein concentration in each erythrocyte lysate sample. HPLC analysis revealed that 5′-AMP levels in *Ampd3^−/−^* erythrocytes were not significantly different from wild type, while ATP and ADP levels of *Ampd3^−/−^* erythrocytes were increased more than 2-fold. In addition, HPLC analysis revealed that the relative levels of adenosine in both wild type and *Ampd3^−/−^* samples were comparable (Figure 3B). The observed rise in ATP and ADP, but not AMP, could suggest that the adenylate equilibrium was favoring the formation of ATP and ADP when the major pathway for 5′-AMP catabolism via AMPD3 was blocked. Alternatively, the rise in ATP levels in *Ampd3^−/−^* erythrocytes could reflect increased glycolysis due to allosteric activation of phosphofructokinase by AMP. Such a possibility would correlate with a decreased level of blood glucose in *Ampd3^−/−^* compared to wild type mice. To address this possibility, fasting blood glucose was measured in the wild type and *Ampd3^−/−^* mice (Figure 3C). Animals were fasted for 12 hr prior to blood glucose measurement. The average fasting blood glucose levels of wild type, *Ampd3^+/−^* and *Ampd3^−/−^* mice were 69 mg/dl, 70 mg/dl, and 73 mg/dl, respectively. The blood glucose levels were not significantly different between the three genotypes. This observation suggests that a decrease in glycolysis is an unlikely explanation. Rather, the elevated ATP and ADP levels likely reflect a reduced degradation of adenine nucleotides in the *Ampd3^−/−^* erythrocytes.

### *Ampd3^−/−^* mice have increased sensitivity to 5′-AMP induced hypometabolism

**Figure 4:**
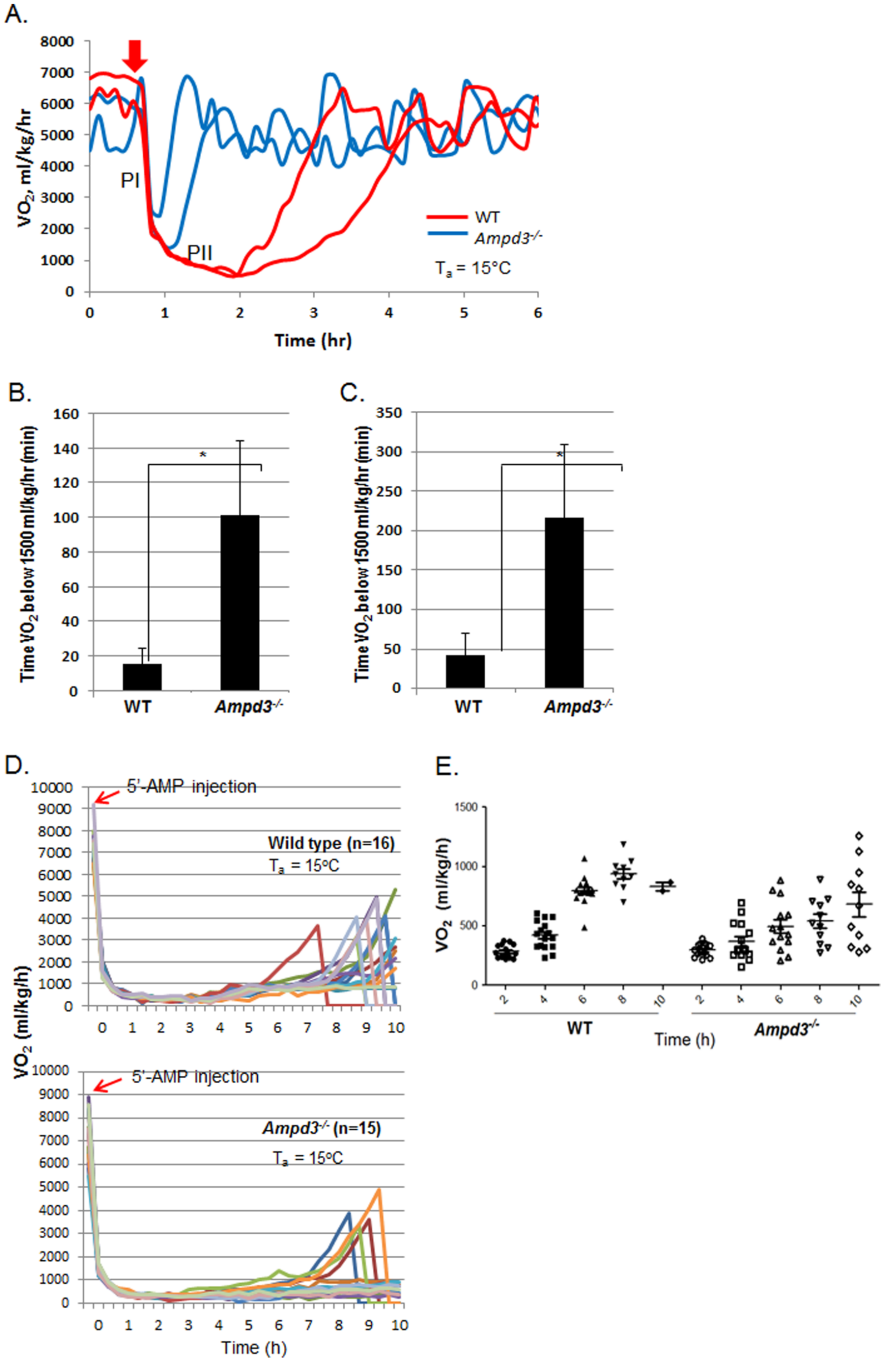
A. *Ampd3^−/−^* mice stay in Phase II much longer than wild type mice when injected (arrow) with a lower dose of 5′-AMP (0.15 mg/g) and maintained at 15°C T_a_. Bar graphs of times that female (B, n = 12) and male (C, n = 3) mice remain in phase II are represented as number of minutes that VO_2_ is below 1500 ml/kg/h. D & E. Trace and time-course quantitative analysis of VO_2_ of wild type (n = 16) and *Ampd3^−/−^* (n = 15) mice given 5′-AMP (0.5 mg/gw) at T_a_ of 15°C, respectively. Note: Mice aroused from deep hypometabolism were removed from CLAMS when their VO_2_ have exceeded 1500 ml/kg/h. Error bars, mean ± SEM.

A major objective for generating the *Ampd3^−/−^* mice was to test our hypothesis that erythrocytes play an important role in mediating 5′-AMP induced hypometabolism. Previously, we reported that, following an injection of 5′-AMP, there are two distinct phases of metabolic reduction [3]. The initial phase I of metabolic rate drop was very rapid and independent of the T_b_ of the animal. During this phase I response, VO_2_ consumption dropped from the euthermic range of 5000–7000 ml/kg/h to about 1500 ml/kg/h within minutes of 5′-AMP administration. This was followed by a more gradual phase II response of metabolic decline that was dependent on T_b_ and was influenced by T_a_. In mice, we observed that phase II corresponds to a VO_2_ below 1500 ml/kg/h. We previously reported that a dosage of 5′-AMP that is less than 0.2 mg/gw produced a phase I but minimal phase II response in the majority of wild type mice at a T_a_ of 15°C [3]. Here, we observed that, with a dose of 0.15 mg/gw 5′-AMP, the majority of wild type mice displayed a phase I metabolic decline but a minimal phase II response (Figure 4A). By contrast, the same dosage given to *Ampd3^−/−^* mice produced both phase I and a significant length of phase II decline. Both male and female *Ampd3^−/−^* mice displayed a similar length of phase II, which was significantly longer than that of wild type siblings of the same sex (Figure 4B, C). These studies show that *Ampd3^−/−^* mice displayed a longer state of hypometabolism when given a dosage of 5′-AMP that was less effective for their wild type siblings.

Next we examined the effect of a higher dosage of 5′-AMP on both wild type and *Ampd3^−/−^* mice. The two genotypes were given 0.5 mg/gw of 5′-AMP and maintained at 15°C T_a_. At 2 h or 4 h post-5′-AMP administration both genotypes were in similar deep hypometabolism with comparable average VO_2_ profiles (Figure 4D, E). At 6 h and 8 h post-administration of 5′-AMP, arousal from deep hypometabolism was evident especially in wild type and their average VO_2_ profile had increased to about 2× that of *Ampd3^−/−^* mice. At 10 h post-5′-AMP administration, only 2 out of 16 wild type compared to 11 out of 15 *Ampd3^−/−^* mice were still in deep hypometabolism. Thus, at a higher 5′-AMP dosage, the average arousal time from deep hypometabolism was significantly delayed in *Ampd3^−/−^* compared with wild type siblings. Together, these mouse genetic studies demonstrate that AMP deaminase 3 plays a major role in modulating the effects of 5′-AMP mediated hypometabolism.

## Discussion

Mammals are warm-blooded and must maintain a relatively constant T_b_ of about 37°C. Under metabolic stress, some mammals are able to decouple euthermic control to undergo torpor or hibernation [1]. During torpor, the drop in T_b_ causes significant slowing of the biochemical and physiological processes allowing the animal to conserve energy and negate the effects of metabolic stress. The biochemical mechanism of natural torpor remains poorly understood. However, it has been noted that the biochemical events that initiate torpor behavior occur rapidly prior to the reduction in T_b_ [1]. Our studies demonstrated that 5′-AMP, when given to mammals, rapidly induced a transient state of hypometabolism that allows for the T_b_ to be safely reduced towards T_a_ [3]. The biochemical mechanism of 5′-AMP induced torpor remains to be conclusively proven. We have previously reported that mice deficient in the extracellular ecto-nucleotidase CD73 have an enhanced response to 5′-AMP for induction of torpor indicating that the extracellular dephosphorylation of 5′-AMP into adenosine is not necessary for the mechanism [3]. We have observed that following 5′-AMP administration, the production of 2,3-BPG is enhanced in the circulation. The critical mutase enzyme for 2,3-BPG synthesis has been identified only in erythrocytes and placenta [16]. Since both male and female mice respond similarly to 5′-AMP induced torpor, the rise in 2,3-BPG implicates the erythrocyte as a cellular target of 5′-AMP. Through isotopic tracking, we and others have observed that erythrocytes readily accumulate radiolabeled 5′-AMP which is converted to ADP using cellular ATP to maintain the adenylate equilibrium [3] [17]. A decrease in erythrocyte ATP has been shown to increase 2,3-BPG production [18]. We proposed that the phase I response, the rapid reduction of VO_2_ following 5′-AMP administration, reflects increased 2,3-BPG allosteric inhibition of hemoglobin oxygen binding. The length of the VO_2_ phase II reflects the uncoupling of thermogenic control allowing T_b_ to cool towards T_a_, thereby reducing the amount of cellular oxygen needed to match its reduced availability. Our proposed mechanism for the erythrocyte's role is supported by observations that hypoxia can induce torpor in pocket mice [19]. In addition, exsanguination is known to be associated with a reduction in T_b_ [20]. Together, these observations support the role of erythrocytes in mediating hypoxia regulation of T_b_.

To test our hypothesis for the erythrocyte's role in torpor, we used mouse genetics to exploit our current understanding of 5′-AMP catabolism. The major catabolism pathway for 5′-AMP is via its deamination to IMP by AMP deaminase. Of the three isozymes of AMP deaminase, AMPD3 is a major isozyme in heart and is the only isozyme in erythrocytes [8] [9]. Our hypothesis predicts that AMPD3 deficiency should confer increased sensitivity to 5′-AMP induced hypometabolism if the erythrocyte is a major mediator of this behavior. We have generated an *Ampd3^−/−^* mouse line with a targeted disruption of the *Ampd3* gene. Our RT-PCR analysis showed that there is no detectable *Ampd3* mRNA in the heart tissue of *Ampd3^−/−^* mice unlike their wild type siblings. Enzymatically, the *Ampd3^−/−^* and wild type sibling mice displayed comparable AMPD activities in all organs except heart and erythrocytes. In the heart, the level of AMP deaminase activity was reduced by 70%. In erythrocytes, the AMPD3 deficient mice displayed no detectable AMP deaminase activity. Further, no detectable activity was observed in serum from *Ampd3^−/−^* blood. Thus, based on enzymatic activity and transcript expression, we have generated mice deficient in AMP deaminase 3. In humans, individuals with a complete lack of AMPD3 are clinically asymptomatic [15]. The erythrocytes of AMPD3 deficient individuals display significantly enhanced steady-state ATP levels. Our analysis of *Ampd3^−/−^* mouse erythrocytes also found elevated levels of ATP and ADP but not 5′-AMP. Recently, an independently derived line of *Ampd3^−/−^* mice was also reported to have greater than two-fold elevated erythrocyte ATP and ADP levels [21]. The study further reported a 3.5-fold elevated level of 5′-AMP that we did not observe in our HPLC analysis. We cannot explain the 5′-AMP level discrepancy between our analysis and those previously reported. We have provided our HPLC analysis trace as evidence to support our conclusion (Figure 3B). We tested the possibility that increased glycolysis could have converted excess 5′-AMP to ADP and ATP. Our analysis revealed that fasting blood glucose levels were not significantly altered and were within the normal physiological range in the AMPD3 deficient mice. Our findings are in line with the report that fasting blood glucose levels of AMPD3 deficient mice were similar to wild type [21]. A possible explanation for our observation is that the adenylate equilibrium preferentially maintains the cellular pool of adenine nucleotides as ATP and ADP rather than 5′-AMP. Typically, adenylate kinase maintains the adenylate equilibrium at a set ratio with ATP levels several fold higher than ADP and at least 10-fold higher than 5′-AMP [22]. In most mammalian erythrocytes, the conversion of 5′-AMP to IMP is irreversible due to the absence of the enzyme adenylosuccinyl synthetase that converts IMP to 5′-AMP [23] [24]. Therefore, the rise in the adenine nucleotide pool in the *Ampd3^−/−^* erythrocytes reflects the loss of a major catabolic pathway for 5′-AMP even when catabolism via dephosphorylation to adenosine remains intact.

The generation of the *Ampd3^−/−^* mice allowed the testing of our hypothesis that erythrocytes are the major target of 5′-AMP in this model of induced hypometabolism. Consistent with our hypothesis, we observed that AMPD3 deficient mice displayed significantly enhanced sensitivity to 5′-AMP for induction of hypometabolism. Using a lower dose that produced only a phase I response in wild type mice, the AMPD3 deficient animals displayed both phase I and phase II responses regardless of gender. Further, when a higher dosage of 5′-AMP was given, the arousal time from deep hypometabolism for the majority of *Ampd3^−/−^* mice was significantly delayed compared with wild type mice. With an intact cellular adenylate equilibrium, we reasoned that the AMPD3 deficient mice undergo 5′-AMP induced hypometabolism with the same basic mechanism as wild type mice. We have previously measured ATP, ADP and 5′-AMP levels of wild type blood taken at various stages of hypometabolism following 5′-AMP administration and observed dynamic changes in the ratio of the three nucleotides over time [3]. Much of the injected 5′-AMP was catabolized and only when 5′-AMP returned to its basal level did the disequlibrium of ATP to ADP ratio return to control levels. We reason that such dynamic changes must also occur in AMPD3 deficient mice but the loss of the major 5′-AMP catabolism pathway slows the recovery of the equilibrium in adenine nucleotide ratios. Consequently, the AMPD3 deficient mice displayed enhanced hypometabolic response to both low and high dosages of 5′-AMP.

Previously it had been proposed that dephosphorylation of 5′-AMP to adenosine and activation of the A_1_ adenosine receptor (A_1_AR) causing bradycardia is an underlying mechanism of 5′-AMP induction of torpor [4]. Our current studies cannot rule out the possibility that decreased AMP deaminase activity in the heart could have contributed to the increased sensitivity of *Ampd3^−/−^* mice to 5′-AMP. Previously, we reported that A_1_AR deficient and wild type mice have similar responses to 5′-AMP as their T_b_ cools at similar rates [3] [25]. Consistent with previous findings, we and others have observed that A_1_AR deficient, unlike wild type mice, did not develop bradycardia following administration of 5′-AMP [26] [25]. These observations argue against an induction of bradycardia by adenosine as the underlying mechanism for 5′-AMP induced torpor.

In summary, we have generated AMPD3 deficient mice and these animals are more responsive to 5′-AMP for induction of hypometabolism. These findings support our proposal that the erythrocyte is a target of 5′-AMP's effect on hypometabolism.
