# Correction: AMP Deaminase 3 Deficiency Enhanced 5′-AMP Induction of Hypometabolism

**DOI:** 10.1371/journal.pone.0091311

**Published:** 2014-03-07

**Authors:** The PLOS ONE Staff

Errors occur in Figure 4A. The labels of the blue line and the red line are switched. The authors have provided a corrected version of Figure 4A here.

**Figure 4A:**
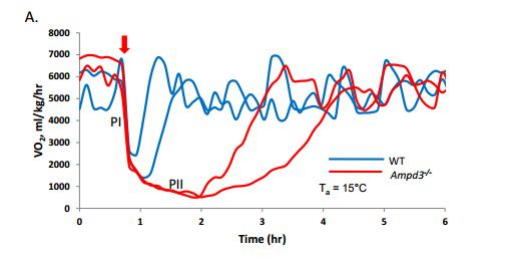
A. *Ampd3^−/−^* mice stay in Phase II much longer than wild type mice when injected (arrow) with a lower dose of 5′-AMP (0.15 mg/g) and maintained at 15°C T_a_. Bar graphs of times that female (B, n  =  12) and male (C, n  =  3) mice remain in phase II are represented as number of minutes that VO_2_ is below 1500 ml/kg/h. D & E. Trace and time-course quantitative analysis of VO_2_ of wild type (n  =  16) and *Ampd3^−/−^* (n  =  15) mice given 5′-AMP (0.5 mg/gw) at T_a_ of 15°C, respectively. Note: Mice aroused from deep hypometabolism were removed from CLAMS when their VO_2_ have exceeded 1500 ml/kg/h. Error bars, mean ± SEM.
